# Pleural myxoid liposarcoma: features of 2 cases and associated literature review

**DOI:** 10.1186/1749-8090-2-48

**Published:** 2007-11-07

**Authors:** Paul Goldsmith, Kostas Papagiannopoulos

**Affiliations:** 1 Department of Thoracic Surgery, St James's University Hospital, Leeds, UK

## Abstract

Primary pleural myxoid liposarcoma is a rare entity and no agreed treatment options have been formulated once diagnosis has been made. We report two cases with subsequent management and make recommendations for treatment pathways in these rare cases.

## Introduction

Liposarcoma is a malignancy arising from fat cells and in adults it accounts for ten to twelve percent of soft tissue tumours. It appears mainly as a retroperitoneal or intra-abdominal mass. However, there are very few cases of primary intrathoracic liposarcomas arising from within the mediastinum. They are usually slow growing with an expansile rather than infiltrative behaviour; hence they present with pressure related symptoms to their neighbouring structures [1]. We report two cases of primary pleural liposarcoma complimented by a literature review.

### Case 1

A 42-year-old male presented to the respiratory physicians with an eight-week history of malaise, left sided upper abdominal pain and increasing shortness of breath. He had an expectorant cough but no haemoptysis, fever, chills and rigors. He was a non-smoker with no significant past medical history.

The chest film [CxR] was suggestive of a large left sided pleural effusion, and a computerised tomogram [CT] confirmed a loculated effusion. A pleural tap was negative for malignancy and a working diagnosis of left empyema was made; the patient was referred to the thoracic department for further management.

At thoracotomy, the left lower lobe was collapsed by a well-encapsulated pedicled mass arising from the para aortic region. A frozen section confirmed the malignant nature of the mass, which was subsequently resected en-bloc and subjected to histology. The patient had an uneventful post operative recovery.

The histological examination revealed a myxoid liposarcoma with some evidence of differentiation. The patient was referred onto the sarcoma team, for adjuvant treatment. At follow up, 2 months after surgery, the patient was making an excellent recovery. The patient however showed evidence of recurrence at his six month follow up and went onto have additional surgery and chemotherapy.

### Case 2

An 80-year-old female presented with increasing shortness of breath over a period of three months, with an exercise tolerance of fifty metres. There was no associated cough, haemoptysis or weight loss. On examination there was decreased air entry on the left side. CxR revealed a large left sided pleural effusion and the CT scan showed a large fluid density mass in the anterior mediastinum. The patient was discussed in a multi disciplinary meeting and proceeded to surgery.

An attempted left VATS was initially performed but this had to be converted to open thoracotomy due to the large nature of the mass. It was eventually excised confirming compression of the normal left lung.

Histology revealed a myxoid liposarcoma and positive resection margins. The patient was deemed not fit for radical radiotherapy and best supportive care was offered. She died eight months after diagnosis from disease progression,

## Discussion

A review of medline revealed a total of six reported cases of primary pleural myxoid liposarcoma [1].

There seems to be a male preponderance [six out of eight] with the mean age at presentation being 55 years. The most common presenting feature is dyspnoea and cough, with an associated pleural effusion in most cases. There is often evident pleuritic pain and weight loss, although asymptomatic cases have been reported at presentation [2,3].

There seems to be no consensus regarding the postoperative management of these patients nor an agreed treatment pathway. Surgery seems to be the most common option, and offers the best chance of cure or disease control in these rare cases. Operative mortality though has been reported to be as high as 40% [4]. Surgical approach does not influence survival.

Pitson *et al *showed a 54% tumour response and reduction after irradiation employing a total of 50gy and 2Gy fractions [4]. Although such responses do not include primary pleural tumours, similar results could potentially be duplicated for preoperative tumour reduction of complex masses or as adjuvant treatment post operatively when positive margins are confirmed. Indeed the case report from Wong *et al *showed that when radiation was offered a longer disease free interval was noted [2].

According to the current available literature, survival varies between 7 months and 8 years. Nader *et al*, reported four patients with primary liposarcoma of which three were myxoid; two died between seven and nine months after initial presentation, with only one survivor at time of publication [1]. Wouters and Greve reported local relapse at four years, which was treated with surgery and adjuvant radiotherapy [5]. This patient remained disease free for a further four years. Evans *et al *reported a case without though mentioning the overall survival period [6]. Wong *et al *published a similar report with the patient alive five months following resection and radiotherapy [2] [Table 1].

**Table 1:** Case reports of primary myxoid liposarcoma of the pleural cavity

**Study**	**Age**	**Symptoms**	**Treatment**	**Survival**
Goldsmith and Papagiannopoulos				
Case 1	42	Malaise, dyspnoea, cough	Resection + radiotherapy. Recurrence – further resection + chemotherapy	Alive at one year
Case 2	80	Dyspnoea	Resection	Died 8 months after Dx
Nader *et al*				
Case 1	45	Bronchitis, pneumonia	Resection + Chemotherapy	Died 7 months after Dx
Case 2	73	Incidental Pleural effusion on CXR	VATS + biopsy, not resected. Palliative care	Died 9 months after Dx
Case 3	80	Incidental finding on CXR	Resection	Unknown
Wouters and Greve	19	None	Resection, radiotherapy four years later	Recurrence at four years, then radiotherapy, in remission
Evans *et al*	61	Chest pain, dyspnoea, pleural effusion	Resection	Unknown
Wong *et al*	38	Cough, dyspnoea	Resection + radiotherapy	Alive at five months

CXR – chest X-ray

Dx – diagnosis

**Figure 1:**
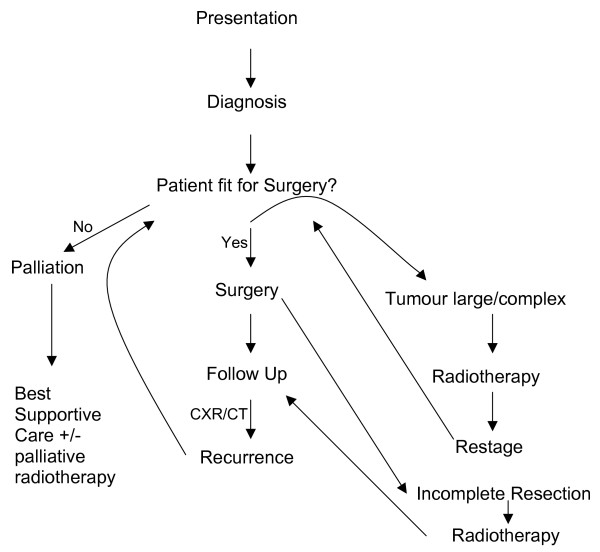
Treatment pathway for myxoid liposarcoma of the pleural cavity.

We have therefore suggested a treatment pathway in patients with primary pleural myxoid liposarcoma based on personal experience and literature evidence [Figure 1].

To conclude myxoid pleural liposarcomas are rare entities and from the existing literature it is difficult to forecast prognostic factors which may correlate with long term survival. Although there is no common consensus, we believe that aggressive surgical management followed by radiotherapy is the best treatment modality. If the tumour is large or complex neoadjuvant radiotherapy could potentially identify responders; these patients could then be offered radical surgical treatment for potential long-term control of the disease.
